# Inpatient Cardiac Rehabilitation after Transcatheter Aortic Valve Replacement Is Associated with Improved Clinical Performance and Quality of Life

**DOI:** 10.3390/jcm10102125

**Published:** 2021-05-14

**Authors:** Pawel Kleczynski, Jaroslaw Trebacz, Maciej Stapor, Robert Sobczynski, Janusz Konstanty-Kalandyk, Boguslaw Kapelak, Krzysztof Zmudka, Jacek Legutko

**Affiliations:** 1 Department of Interventional Cardiology, Institute of Cardiology, Jagiellonian University Medical College, John Paul II Hospital, 31-202 Krakow, Poland; jartrebacz@gmail.com (J.T.); maciej.stapor@gmail.com (M.S.); zmudka@icloud.com (K.Z.); jacek.legutko@uj.edu.pl (J.L.); 2 Department of Cardiac and Vascular Surgery and Transplantology, Institute of Cardiology, Jagiellonian University Medical College, John Paul II Hospital, 31-202 Krakow, Poland; sobczynski@poczta.fm (R.S.); jakonstanty@poczta.onet.pl (J.K.-K.); bogus.kapelak@gmail.com (B.K.)

**Keywords:** aortic valve stenosis, cardiac rehabilitation, frailty, transcatheter aortic valve replacement

## Abstract

Cardiac rehabilitation (CR) provides multifactorial support and intervention for cardiac patients and improves quality of life (QoL). We aimed to assess clinical performance and QoL changes in patients undergoing transcatheter aortic valve replacement (TAVR) scheduled directly to inpatient CR (CR group) and those who were discharged home (DH group). The following patient-related outcomes were recorded: 5 m walk time (5MWT), 6 min walk test (6MWT), handgrip strength (HGS) with dynamometer, Katz index of Independence of Activities in Daily Living (KI of ADL), Hospital Anxiety and Depression Scores (HADS) Score. Quality of life was evaluated with Kansas City Cardiomyopathy Questionnaire (KCCQ). Baseline data, 30-day and 6- and 12-month data were assessed. The CR group consisted of 52 patients and 53 were in the discharged home (DH group). When we compared outcomes between the groups, the 5MWT, 6MWT, HGS KI of ADL, and KCCQ were significantly better in the CR group at 30 days (*p* = 0.03, *p* = 0.01, *p* = 0.02, *p* = 0.048, respectively), and no difference was found in HADS scores. At 6 months, the effect of CR was sustained for 6MWT, HGS, KI of ADL, and KCCQ (*p* = 0.001, *p* = 0.001, *p* = 0.03, *p* = 0.003, respectively) but not for 5MWT. Interestingly, at 12 months, the CR group had better performance only in 6MWT and HGS compared with the DH group (*p* = 0.04, *p* = 0.03, respectively). We showed that inpatient CR is strongly associated with better clinical performance and QoL in patients undergoing TAVR. All patients may benefit from CR after TAVR. The most important aspect of inpatient CR after TAVR from the patient’s perspective may be better performance in daily activities; however, performance was attenuated after 1 year.

## 1. Introduction

Transcatheter aortic valve replacement (TAVR) has emerged as a therapy option for older high-risk symptomatic patients presenting with severe aortic valve stenosis (AS) and is currently extending its indications toward intermediate- and low-risk subjects [1,2,3]. It has been previously reported that TAVR improves not only survival but also quality of life (QoL) after careful clinical selection and preprocedural workup of patients with AS [4,5,6]. Patients after a successful procedure are transported to another hospital due to many comorbidities or scheduled for cardiac rehabilitation (CR) or even discharged home, despite being invited to a CR program. Cardiac rehabilitation provides multifactorial support and intervention for cardiac patients and, moreover, improves QoL, reduces depression, and has been demonstrated to be a factor that reduces hospital readmissions and mortality [7,8]. The data showing the impact of inpatient CR on daily activity and overall performance of TAVR patients during 1 year of follow up are scarce. In our study, we aimed to assess clinical performance and QoL changes in patients undergoing TAVR scheduled directly to inpatient CR (CR group) and those who were discharged home (DH group).

## 2. Materials and Methods

### 2.1. Patients and Study Design

We included 105 consecutive patients undergoing TAVR via transfemoral route between January 2017 and March 2018 who agreed to participate in the study. The study protocol conformed with the ethical guidelines of the 1975 Declaration of Helsinki with later amendments. The study was a retrospective registry set in a single center and the institutional ethical board was informed. All patients gave written, informed consent. Demographics, comorbidities, and treatment data were collected at baseline. Transcatheter aortic valve replacement procedures were performed with conscious sedation in all patients. Only Edwards Sapien 3 (Edwards Lifesciences, Irvine, CA, USA) and Evolut R (Medtronic Inc., Minneapolis, MN, USA) prostheses were used. Discharged home patients were, however, eligible for inpatient CR but refused to take part in the CR program due to various reasons (e.g., social factors, presence of caregiver at home, distance from home, etc.) and stayed only in ambulatory control. They were also advised to implement a “sit to stand” activity at home every day, if possible.

### 2.2. Assessment of Clinical Performance

Study outcomes were assessed at baseline (ca. 2–3 months before index procedure), 30 days, 6 months, and 12 months. The following patient-related outcomes were recorded (frailty indices): 5 m walk time (5MWT) [9], 6 min walk test (6MWT) [10], handgrip strength (HGS) with dynamometer (Kern Map 80K1S, Kern & Sohn GmbH, Balingen, Germany), Katz index of Independence of Activities in Daily Living (KI of ADL, 6 points—not frail, <6 points—frail) [11], Hospital Anxiety and Depression Scores (HADS, 0–7 normal, 8–10 borderline, 11–21 abnormal) Score [12]. QoL was evaluated with the Kansas City Cardiomyopathy Questionnaire (KCCQ) [13]. The 5 m walk time (5MWT) was assessed by the same physician each time at the cardiology ward using a standard stopwatch, and the distance of 5 m was estimated with a retractable counter and precisely marked. The test was performed three times and we present the mean result of the three trials. The 6 min walk test (6MWT) was supervised by a trained study nurse using a stopwatch for time assessment and measuring the overcome distance on a 30-m stretch of unimpeded walkway by the patient. Two cones marked the distance that needed to be covered. Hand grip strength assessment was performed using the patient’s dominant hand and patients were asked to squeeze the hand as tightly as possible (repeated twice).

### 2.3. Cardiac Rehabilitation Protocol

Cardiac rehabilitation was implemented immediately after hospital discharge as a 14-night stay with 6 days a week of programming at an inpatient CR facility. A rehabilitation program was prescribed for each patient based on data gathered from their functional capacity test with the modified Bruce with ramp protocol (expressed as multiples of resting metabolic equivalent (METS)) and requesting their individual goals. The individualized ramp protocol was designed based on age, gender, and weight. The predicted values of VO_2max_ (mL/min) were calculated using the formulas: (50.72 − (0.372 × age)) × weight × 1.1 for men and (22.78 − (0.17 × age)) × (weight + 43) × 1.1 for women [14]. The predicted peak exercise capacity in MET was calculated by dividing the received values by (weight × 3.5). The participants started walking on the treadmill at 1.6 km/h and an incline of 0 percent. The speed and treadmill incline linearly increased until their maximum values were achieved. The maximum speed was chosen to be 3.2 km/h. The maximum treadmill incline was 5%.

Exercise prescription consisted of cardiovascular training and resistance training using a cardiovascular treadmill (walk) (AspelB612, Aspel SA, Zabierzów, Poland) and cycloergometer (with no resistance) (AspelCRG200, AspelSA) as well as functional exercise such as “sit to stand to sit”. The exercise intensity and volume were progressively increased based on the self-reported Borg scale of perceived exertion [15]. Duration of each session started at 15 min and lasted up to 30 min on the last day of CR stay.

Psychosocial intervention during inpatient CR stay included motivation and stress relief and was performed by a qualified nurse or physiotherapist.

Lifestyle modification included adherence to hospital discharge recommendations.

The cardiac rehabilitation program was supervised by a physician, nurse, and a physiotherapist. Patients were invited to inpatient CR only once during the follow-up period.

### 2.4. Statistical Analysis

Continuous variables are expressed as median (interquartile range) and categorical variables are expressed as number (percentage). Normality was checked by the Shapiro–Wilk test. Continuous variables were compared by unpaired and paired Student’s *t* tests when normally distributed and by the Mann–Whitney U test or Wilcoxon signed-rank test when not normally distributed, as appropriate. The Pearson’s χ^2^ test or Fisher’s exact test was used to compare the category frequencies. Repeated measures between–within parameters were assessed with ANCOVA with adjustment for baseline scores. All tests were two-tailed, and a *p* value <0.05 was considered statistically significant. Statistical analysis was performed using STATISTICA 13.3 (TIBCO Software Inc., Palo Alto, CA, USA).

## 3. Results

### 3.1. Clinical Data

The CR group consisted of 52 patients and 53 were discharged home (DH group). There was no difference in baseline clinical and echocardiographic data between study groups (Table 1). Mean postprocedural transaortic pressure gradient was similar in the CR and DH group (8.5 ± 2.7 vs 9.2 ± 2.4 mmHg, *p* = 0.21) as well as left ventricle ejection fraction (50.0 ± 4.5 vs 49.9 ± 4.1%, *p* = 0.32, respectively). Median METS at discharge in the CR group was 3.7 (3.5–4.9) vs 3.8 (3.3–5.10 in the DH group (*p* = 0.54). Mean hospital stay did not vary between both groups (7.1 ± 2.5 vs 7.5 ± 2.1 days in the CR and DH group, *p* = 0.84). We noted following reasons for CR decline: social factors (*n* = 9, 17.0%), presence of caregiver at home (*n* = 12, 22.6%), distance from home (*n* = 19, 35.8%), and family problems (*n* = 13, 24.5%). During 12-month follow-up all-cause death rates were similar (7.7% in the CR group vs 7.5% in the DH group, *p* = 0.91), bleeding complications requiring blood transfusion occurred in 13.4% in the CR and 15.0% in the DH group (*p* = 0.12), pacemaker implantation was required in 4 (7.7%) patients in the CR group and in 5 (9.4%) patients in the DH group (*p* = 0.36).

### 3.2. Patients’ Performance Measures

All patients completed the CR program. Detailed study outcomes are presented in Table 2. During CR the median score of the Borg Scale was 14.5 [11.0–17.0]. We found interesting outcomes in the CR group compared with the DH group depending on time-point of clinical follow-up. Obviously, outcomes within the groups were better at each time point after the index procedure compared with baseline assessment (*p* <0.001 for all study outcomes). However, when we compared outcomes between the groups, the 5MWT, 6MWT, HGS, KI of ADL, and KCCQ were significantly better in the CR group at 30 days (*p* = 0.03, *p* = 0.01, *p* = 0.02, *p* = 0.048, and *p* = 0.04, respectively), and no difference was found in HADS scores. At 6 months, the effect of CR was sustained for 6MWT, HGS, KI of ADL, and KCCQ (*p* = 0.001, *p* = 0.001, *p* = 0.03, *p* = 0.03, respectively) but not for 5MWT. Interestingly, at 12 months the CR group had better performance only in 6MWT and HGS compared with the DH group (*p* = 0.04, *p* = 0.03, respectively).

## 4. Discussion

In this study, we showed that inpatient CR is strongly associated with better clinical performance and QoL in patients with severe AS undergoing TAVR. All patients, even if considered as frail, may benefit from inpatient CR after TAVR. The most important aspect of CR after TAVR from the patient’s perspective may be better outcomes in daily activities; however, outcomes were attenuated after 1 year. The longer time interval from the end of inpatient CR, the weaker the performance of patients was noticed. This may raise a question about whether CR should be offered to TAVR patients periodically after discharge and then during follow-up to allow the CR effect to last longer. So far, no recommendations of working groups of the European Society of Cardiology are provided for patients after TAVR.

Establishing an effective program of CR for older patients after TAVR is a very complex task, requiring cooperation between physicians, nurses, physiotherapists, dieticians and, sometimes, social workers. Physicians should have an adequate expertise in cardiology, geriatrics, and various aspects of physical rehabilitation of the octogenarian. Drug administration may also deviate in the course of rehabilitation compared with that at discharge and must be considered based on physical activity and, for example, blood pressure. It is quite common for patients after TAVR to add more hypertensive drugs. Additionally, echocardiographic assessment may be required during the CR program. Moreover, physiotherapists should also be trained and should become familiar with how to work with such a subset of patients who always present with different accompanying comorbidities. Effective CR in older cardiac patients may be attained only with a multidisciplinary approach and with attention to the wellness of other non-cardiac organs and systems, for example musculoskeletal system. Inpatient cardiac rehabilitation is believed to enhance physical and functional performance after TAVR, but all predictors are usually assessed based on baseline clinical characteristics of patients before TAVR without regard to CR referral.

In a study by Tarro Genta et al. the authors showed that in TAVR patients who underwent CR, lower exercise tolerance, higher Barthel Index, and serum creatinine level at discharge from CR may predict 3-year mortality [8]. In our study we did not find, however, association of poorer performance with mortality after TAVR, probably due to the relatively short follow-up period. However, QoL was strongly affected, especially in daily activities. In a recent meta-analysis of six CR studies by Anayo et al., efficacy, safety, and costs of CR after TAVR and aortic valve surgery were evaluated [16]. Exercise-based CR improved exercise capacity of post-TAVR and post-aortic valve surgery patients in the short term (2–12 months). Data on other outcomes including QoL and clinical events were limited. Findings from our study may complement that research, contributing more data regarding QoL. In a study by Zanettini et al., 60 patients referred for CR after TAVR underwent in-hospital and post-discharge multidimensional assessments with various measures to evaluate clinical, functional, and nutritional status, degree of autonomy, cognitive impairment, depression, and quality of life [17]. During a CR program, most patients showed significant improvement, which remained stable in the majority of subjects during mid-term follow-up (540 days), which was contrary to our results. In our study, no nutritional evaluation or support was performed during cardiac rehabilitation. Pressler et al. showed in a randomized study that exercise training was safe and effective with respect to improvements in exercise capacity, muscular strength, and quality of life in patients after TAVR [18]. However, the CR program lasted 8 weeks, and the outcomes were assessed at that timepoint only, with no longer-term follow-up.

In another meta-analysis by Ribeiro et al., again, the CR program after TAVR versus surgical aortic valve replacement was compared, yielding comparable outcomes in both groups with similar benefits [19]. However, the assessment of benefit was evaluated only once, directly after completion of the CR program, contrary to our study in which the outcomes were measured after 1, 6, and 12 months after the index procedure.

An aspect of patients not willing to participate in inpatient CR should be mentioned. Patients who decline CR are different from those willing to participate, not in clinical characteristics but in behavioral or socioeconomical aspects, which may play an important role [20]. In our study following reasons for declining CR were observed: social factors, the presence of a caregiver at home, the program’s distance from home, and family problems. Home-based CR, on the other hand, is believed to be better in terms of long-term maintenance of rehabilitation in patients with cardiac disease [21,22], yet the real level of compliance and adherence in the elderly (>80 years) is difficult to determine.

### Study Limitations

The study was conducted in a single center setting with a relatively small sample size, but it presents real-world outcomes data of consecutive patients who agreed to participate in the study. Moreover, the study was not randomized, allowing stronger conclusions to be drawn; consequently, the results may be biased—a fact that should be considered during interpretation of the findings. Rogers et al. showed that it is possible to enroll TAVR patients into a randomized clinical trial in their pilot study [7]. However, no data have been published so far by his team. Real compliance with home rehabilitation of patients who were discharged home remains unknown.

## 5. Conclusions

We showed that inpatient CR is strongly associated with better clinical performance and QoL in patients undergoing transcatheter aortic valve replacement. All patients may benefit from CR after transcatheter aortic valve replacement. The most important aspect of CR after TAVR from the patient’s perspective may be better performance of daily activities; however, performance was attenuated after 1 year.

## Figures and Tables

**Table 1:** Baseline clinical and echocardiographic characteristics.

Variable	All (*n* = 105)	Cardiac Rehabilitation Group (*n* = 52)	Discharged Home Group (*n* = 53)	*p* Value
Age, mean ± SD (years)	80 ± 4.5	81 ± 4.9	80 ± 5.5	0.52
Age ≥80 years	24 (23%)	13 (25%)	11 (21%)	0.48
Men	42 (40%)	22 (42%)	20 (38%)	0.29
Body mass index, median (IQR) (kg/m^2^)	25.1 (23.7–27.6)	23.9 (22.4–27.9)	24.2 (23.0–28.3)	0.73
Estimated glomerular filtration rate, median (IQR) (mL/min/1.73 m^2^)	52 (39.5–77.8)	55 (39.9–75.1)	54 (40.1–77.5)	0.63
NYHA class				
I + II	0	0	0	0.49
III	76 (72%)	39 (75%)	37 (70%)	
IV	29 (28%)	14 (27%)	15 (28%)	
Arterial hypertension	96 (91%)	47 (90%)	49 (92%)	0.25
Diabetes mellitus	47 (45%)	22 (42%)	25 (47%)	0.34
Atrial fibrillation	28(27%)	13 (25%)	15 (28%)	0.29
Previous myocardial infarction	39 (37%)	19 (37%)	20 (43%)	0.53
Previous percutaneous coronary intervention	37 (35%)	18 (35%)	19 (36%)	0.31
Previous coronary artery bypass grafting	15 (14%)	7 (14%)	8 (15%)	0.32
Chronic obstructive pulmonary disease	18 (17%)	10 (19%)	8 (15%)	0.13
Peripheral artery disease	25 (24%)	12 (23%)	13 (24%)	0.29
Stroke/transient ischemic attack	18(17%)	8 (15%)	10 (19%)	0.21
Pacemaker	11 (10%)	6 (11%)	5 (9%)	0.72
Logistic Euroscore II, median (IQR)	9.9 (7.8–13.3)	10.2 (7.9–13.5)	9.9 (7.7–12.9)	0.62
The Society of Thoracic Surgeons score, median (IQR)	8.2 (6.2–10.1)	8.5 (6.1–10.5)	7.9 (6.0–11.2)	0.19
Maximal transaortic gradient, mean ± SD (mmHg)	82 ± 12.9	82 ± 16.5	84 ± 14.3	0.36
Mean transaortic gradient, mean ± SD (mmHg)	44 ± 4.5	45 ± 5.1	43 ± 6.2	0.31
Aortic valve area, mean ± SD (cm^2^)	0.7 ± 0.5	0.71 ± 0.3	0.72 ± 0.4	0.55
Left ventricle ejection fraction, mean ± SD (%)	50 ± 6.9	49 ± 9.4	50 ± 6.8	0.27
Edwards Sapien 3	41 (39%)	21 (40%)	20 (38%)	0.78
Evolut R	64 (61%)	31 (60%)	33 (62%)	0.39

IQR, interquartile range; NYHA, New York Heart Association functional class; SD, standard deviation.

**Table 2:** Study outcomes at baseline, 30 days, 6 months, and 12 months.

Variable	Cardiac Rehabilitation Group (*n* = 52)	Discharged Home Group (*n* = 53)	*p* Value
Outcomes at baseline			
5MWT, mean ± SD (seconds)	6.8 ± 1.2	6.9 ± 1.3	0.86
6MWT, mean ± SD (meters)	295 ± 42	283 ± 39	0.84
HGS, mean ± SD (kg)	26 ± 12.5	28 ± 13.1	0.76
KI of ADL, mean ± SD (points)	4.7 ± 1.2	4.6 ± 1.1	0.51
HADS Anxiety, median (IQR) (points)	6 (3–7)	7(4–8)	0.12
HADS Depression, median (IQR) (points)	2 (1–5)	2(1–4)	0.92
KCCQ, mean ± SD (points)	72.1 ± 21.1	73.5 ± 19.4	0.15
Outcomes at 30 days			
5MWT, mean ± SD (seconds)	5.1 ± 0.9	5.8 ± 1.3	0.03
6MWT, mean ± SD (meters)	397 ± 24	384 ± 29	0.01
HGS, mean ± SD (kg)	36 ± 9.3	28 ± 12.1	0.02
KI of ADL, mean ± SD (points)	5.0 ± 0.8	4.8 ± 1.0	0.048
HADS Anxiety, median (IQR) (points)	2 (1–5)	2 (1–6)	0.82
HADS Depression, median (IQR) (points)	1 (1–3)	1(1–4)	0.12
KCCQ, mean ± SD (points)	82.5 ± 15.0	76.5 ± 12.4	0.04
Outcomes at 6 months			
5MWT, mean ± SD (seconds)	5.8 ± 1.0	5.9 ± 1.1	0.36
6MWT, mean ± SD (meters)	426 ± 34	392 ± 19	0.001
HGS, mean ± SD (kg)	38 ± 7.3	30 ± 10.1	0.001
KI of ADL, mean ± SD (points)	5.0 ± 0.8	4.7 ± 1.2	0.03
HADS Anxiety, median (IQR) (points)	2 (1–5)	2 (1–5)	0.94
HADS Depression, median (IQR) (points)	1 (1–3)	1 (1–4)	0.17
KCCQ, mean ± SD (points)	86.5 ± 17.0	78.5 ± 11.4	0.03
Outcomes at 12 months			
5MWT, mean ± SD (seconds)	6.1 ± 0.9	6.3 ± 1.1	0.08
6MWT, mean ± SD (meters)	410 ± 22	389 ± 18	0.04
HGS, mean ± SD (kg)	35 ± 10.5	30 ± 11.6	0.03
KI of ADL, mean ± SD (points)	5.1 ± 0.6	4.9 ±1.0	0.19
HADS Anxiety, median (IQR) (points)	2 (1–4)	2 (1–4)	0.92
HADS Depression, median (IQR) (points)	1 (1–3)	1 (1–4)	0.76
KCCQ, mean ± SD (points)	80.1 ± 18.5	77.4 ± 17.4	0.17

5MWT, 5m walk time; 6MWT, 6 min walk test; HGS, hand grip strength; KI of ADL, Katz index of Independence of Activities in Daily Living; HADS, Hospital Anxiety and Depression Scores; KCCQ, Kansas City Cardiomyopathy Questionnaire.

## Data Availability

The data presented in this study are available on request from the corresponding author.
